# Research on Inner Gas Inflation Improvements in Double-layer Gas-assisted Extrusion of Micro-tubes

**DOI:** 10.3390/polym12040899

**Published:** 2020-04-13

**Authors:** Cheng Luo, Xingyuan Huang, Tongke Liu, Hesheng Liu

**Affiliations:** 1 College of Mechanical and Electrical Engineering, Nanchang University, Nanchang 330031, China; luocheng@ncu.edu.cn (C.L.); 405928917131@email.ncu.edu.cn (T.L.); 2 School of Chemical Biology and Materials Science, East China University of Technology, Nanchang 330031, China; hsliu@vip.163.com

**Keywords:** micro-tube formation, gas-assisted extrusion(GAE), double gas layer, die design, forced exhaust flow rate, numerical calculation

## Abstract

Micro-tubes have small diameters and thin wall thicknesses. When using double-layer gas-assisted extrusion (DGAE) technology to process micro-tubes, due to the influence of flow resistance, airflow from the inner gas-assisted layer cannot flow into the atmosphere through the lumen. Over time, it will inflate or even fracture the micro-tubes intermittently and periodically. To solve this problem, a new double-layer micro-tube gas-assisted extrusion die was designed in this study. Its mandrel has an independent airway leading to the lumen of the extrudate, with which the gas flow into the lumen of the extrudate can be regulated by employing forced exhaust. Using the new die, we carried out extrusion experiments and numerical calculations. The results show a significant positive correlation between micro-tube deformation and gas flow rate in the lumen of a micro-tube. Without considering the refrigerant distortion of the microtube, the flow rate of forced exhaust should be set equal to that of the gas from the inner gas-assisted layer flow into the micro-tube lumen. By doing this, the problem of the micro-tube being inflated can be eliminated without causing other problems.

## 1. Introduction

With the development of catheter-based diagnosis and treatment technology, plastic micro-tubes are increasingly being used in clinical medicine for applications such as drainage, perfusion, and the auxiliary introduction of instruments [1,2,3,4,5,6]. As most plastic micro-tubes are produced by extrusion, researchers have studied different aspects of micro-tube extrusion including die design [7,8,9,10], process parameters [11,12,13,14], and numerical simulations [15,16,17]. 

Due to the viscoelasticity of plastic melt, some problems are likely to occur during extrusion processing [18,19,20,21]. The melt is subjected to tensile and shear deformations in the die. Only some of these deformations are relaxed in the die, and the rest are elastically restored after being squeezed out of the die, which results in die swell. When the extrusion rate is high, elastic turbulence can easily occur, leading to unstable flow and eventually melt fracture. To reduce the negative impact of these problems on product quality and production rates, a variety of solutions have been studied by scholars, for example, lubrication extrusion [22,23,24], low-friction coated die extrusion [25,26], vibration-assisted extrusion [27,28,29,30], and gas-assisted extrusion [31,32,33].

Among these methods, the GAE process was first reported by Liang in 2001 [31]. He injected gas into a metal die–molten polymer interface with a tailor-made die and established a stable gas layer at the interface, which was able to give rise to full slip extrusion of the melt. Follow-up studies by other scholars confirmed that GAE is a promising method for improving product quality and production efficiency. Further, it also helps to reduce energy consumption because the die pressure drop in gas-assisted extrusion is significantly smaller than that in traditional extrusion [34,35,36,37,38,39,40,41].

**Figure 1:**
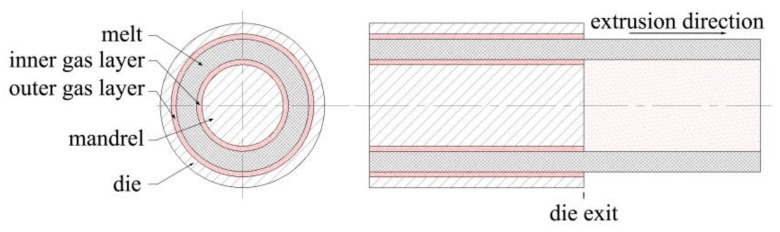
Schematic illustration of double-layer gas-assisted extrusion formation.

**Figure 2:**
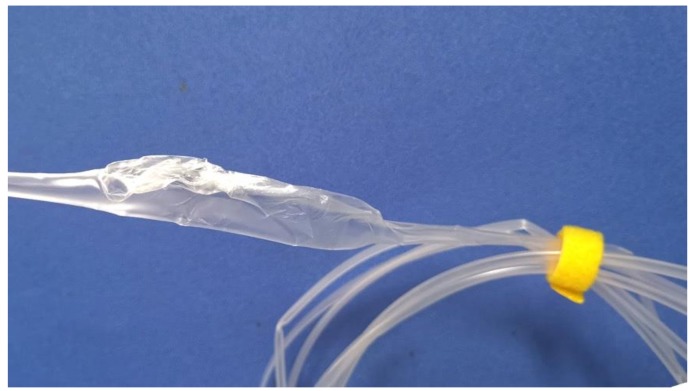
Polypropylene (PP) micro-tube with swelling and fracture.

Based on previous work conducted by his research group [32,42,43,44,45,46,47], Ren expanded the application scope of GAE technology by applying double-layer gas-assisted extrusion technology to process a micro-tube [48,49]. Its formation principle is shown in Figure 1. By controlling the gas injection parameters, a very thin but stable air cushion is formed at the die–melt interface and melt–mandrel interface; these areas are named the inner gas layer and outer gas layer, respectively. The air cushions give rise to essentially full slip wall boundary conditions, and then the polymer melt can be extruded from the die as plug flow. Experimental results indicate that DGAE can achieve its expected goal to a certain extent—it can eliminate die buildup as well as die swell on the inner and outer walls of the micro-tube; it also eases instability in polymer melt flow during high-speed extrusion. However, there is a new problem that needs to be resolved: Due to the influence of flow resistance, airflow from the inner gas-assisted layer cannot flow into the atmosphere through the lumen. Over time, in the downstream area near the die exit it will inflate or even fracture the micro-tubes intermittently and periodically. This type of fracture occurs periodically, making it impossible to obtain a sufficiently long micro-tube with a constant radial dimension in the experiment, as shown in Figure 2.

This study is a continuation of Ren’s work, and the goal is to find an effective method to avoid swelling or rupture during the process of micro-tube extrusion using double-layer gas-assisted extrusion technology. It includes two aspects. First, to meet the unique needs of gas flow control, a new DGAE die is designed and manufactured, and the DGAE experimental system supporting it is rebuilt. Second, based on the new die, DGAE experiments and numerical calculations are performed. By combining the experimental and simulation results, the influence of the gas flow rate in the lumen on micro-tube deformation is analyzed to find a proper solution to the problem.

## 2. Materials and Methods

### 2.1. Experimental Methods

#### 2.1.1. Materials

In the experiments, the molten polymer extruded was an extrusion-grade polypropylene (PP, type: PPH-T03 without any processing aids or additives) supplied by SINOPEC Co., Beijing, China. Its property values are shown in Table 1.

#### 2.1.2. Experimental apparatus

**Figure 3:**
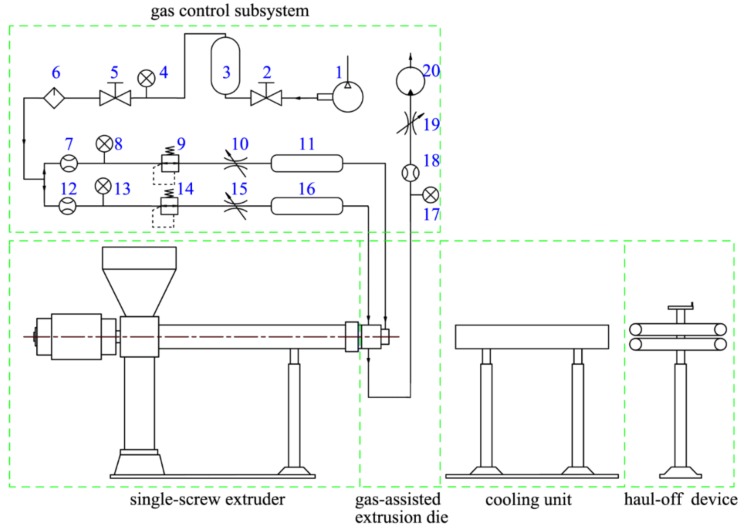
Schematic diagram of the gas-assisted extrusion (GAE) forming system. 1—Air compressor; 2,5—Switch valve; 3—High-pressure gas storage tank; 4,8,13,17—Pressure gauge; 6—Dry filter; 7,12,18—Rotameters; 9,14—Pressure-adjustable valve; 10,15,19—Throttle valve; 11,16—Gas heating device; 20—Vacuum pump.

The schematic diagram of the GAE forming system, which was used to carry out extrusion experiments, is shown in Figure 3. It consists of a single screw extruder, GAE die, a cooling unit, a haul-off device, and a gas control subsystem. More details of the experimental apparatus are depicted in Table 2.

Compared with traditional extrusion, the gas control subsystem is unique to DGAE systems. It provides gas-assisted die with two dry air streams whose flow rate and temperature can be controlled independently, and it is also responsible for timely exhaust of excess gas from the micro-tube lumen. More concretely, in the gas control system, the gas from the air compressor is divided into two independent air streams after passing through the high-pressure gas storage tank and filter dryer. Then, they flow through the rotameters, the adjustable pressure valve, and the throttle valve, respectively, before moving into the gas heating device. After being heated to a predetermined temperature, they flow into the inlet of the inner gas layer channel and outer gas layer channel on the DGAE die, respectively.

**Figure 4:**
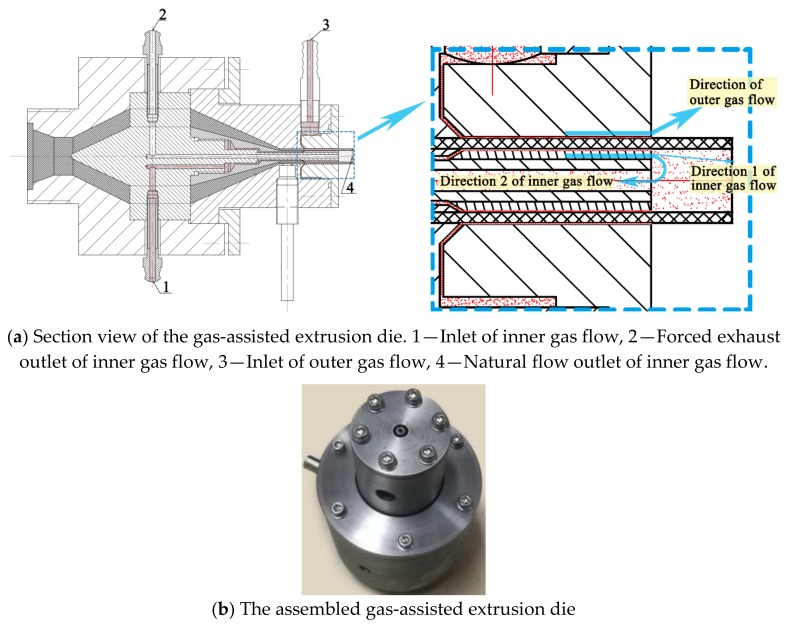
Double-layer gas-assisted extrusion die.

In these experiments, the die was one of the most important apparatuses, and its design needed to be combined with specific experimental requirements. It was found that in the process of DGAE, the flow rate required to establish a stable inner gas cushion is far higher than what could be expelled from the lumen of the micro-tube in time, which caused the expansion and rupture of micro-tubes in the downstream area near the die exit. Based on this analysis, we designed a new die structure with three utterly independent gas channels, as shown in Figure 4. In this structure, after the two branches of the gas from the gas control system enter the die, they flow along their respective channels, one reaching the die–melt interface to form the outer gas layer and the other reaching the melt–mandrel interface to form the inner gas layer. In the end, the gas from the outer gas layer flows out into the atmosphere directly. Meanwhile, after the gas from the inner gas layer flows out of the die, part of it flows naturally into the atmosphere through the lumen along direction 1, and the rest flows in direction 2 and is pumped into the atmosphere by a vacuum pump through the independent airway in the mandrel.

#### 2.1.3. Extrusion

The brief experimental procedure is as follows:

(1) Add PP particles into the extruder hopper.
(2) Turn on the power of each heating device in the GAE system and set the corresponding temperature parameters.
(3) Turn on the power switch of the air compressor, and then set the airflow parameters by adjusting each gas control element in the gas path, and inject assisted-gas into the die.
(4) After the temperature of the gas heater, extruder barrel, and extrusion die reach the set values, start the extruder and carry out the extrusion experiment.

Gas-assisted extrusion experiments were performed four times. In these experiments, except for the different flow rates evacuated through the forced exhaust outlet of the inner gas flow (hereinafter referred to as the forced exhaust flow rate), other experimental parameters remained unchanged. The parameters involved in the experiments are listed in Table 3. 

### 2.2. Numerical Simulation

Numerical simulations were carried out using POLYFLOW, a commercial finite element computational fluid dynamics package [50]. They were used to predict the physical field distributions in the melt and gas while they were flowing through the die, and flowing through the outside zones of the die.

#### 2.2.1. Calculation Domain and Grid Generation

**Figure 5:**
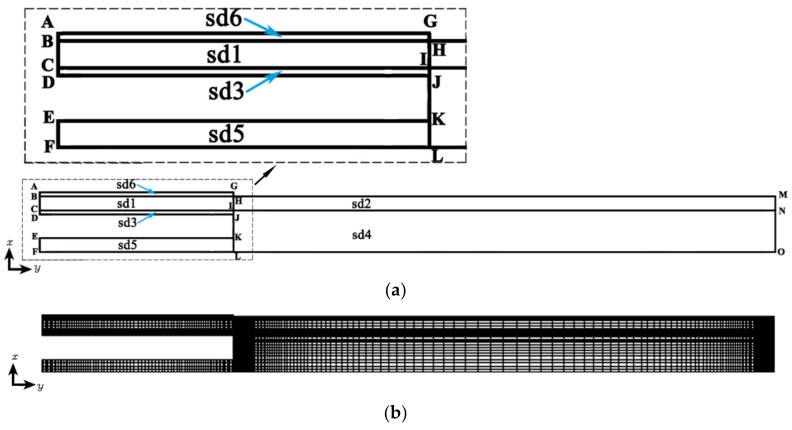
Computational domain and meshing results. (**a**) Computational domain and its boundaries; (**b**) meshing results.

In view of the rotational symmetry of the single-lumen micro-tube and the boundary conditions on it, half of its axial section was selected for numerical calculation, and the terminal line selected for calculation was located downstream of the die exit and 0.051 m away from the die exit. The computational domain obtained for simulations based on this is shown in Figure 5a. It contained six subdomains: sd1 (area enclosed by points B,H,I,C), sd2 (area enclosed by points H,M,N,I), sd3 (area surrounded by points C,I,J,D), sd4 (area enclosed by points I,N,O,L), sd5 (area enclosed by points E,K,L,F), and sd6 (area enclosed by points A,G,H,B), where sd1 and sd2 are melt regions, sd3, sd4, and sd5 are inner gas layer regions, and sd6 is an outer gas layer region. In this calculation domain, the lengths of the boundaries were as follows: *l_AB_* = *l_CD_* = 1 × 10^−4^ m, *l_BC_* = 1.4 × 10^−3^ m, *l_EF_* = 1 × 10^−3^ m, *l_AG_* = 1.8 × 10^−2^ m, *l_FO_* = 6.9 × 10^−2^ m, *l_JK_* = 1.9 × 10^−3^ m.

The grid was created in Gambit using a structured grid with quadrilateral cells, and the grid resolution was increased at the die exit zone as well as at the interface area of gas and melt, as shown in Figure 5b. Consequently, the mesh for the calculation domain had 10,000 cells.

#### 2.2.2. Basic Hypotheses 

To save computer time and to simplify calculations, based on the relative theories, the following hypotheses were made for the numerical simulations:

(1) The PP melt is an incompressible non-Newtonian viscoelastic fluid [48], and the gas is a compressible Newtonian fluid.
(2) Under the parameters set in this study, the Reynolds number of the gas is less than the turbulence standard, and the turbulent flow of the gas is ignored. Both the PP melt and the gas are regarded as non-isothermal steady-state laminar flows.
(3) The effects of inertial force and gravity are ignored, as PP melt has a high viscosity and low flow velocity, while the density and viscosity of the gas are small.
(4) The relative sliding of the gas and inner surface of the die as well as the gas and the PP melt surface are ignored.

#### 2.2.3. Governing Equations

Based on CFD theory, the governing equations for viscoelastic fluids take the following form:

(1) $$\mathrm{Continuity}\;\mathrm{equation}:\frac{D\rho }{Dt}+\rho \left(\nabla \cdot v\right)=0$$

(2) $$\mathrm{Momentum}\;\mathrm{equation}:\rho \frac{Dv}{Dt}=\rho g-\nabla p+\nabla \cdot \tau$$

(3) $$\mathrm{Energy}\;\mathrm{equation}:\rho C_{v}\frac{DT}{Dt}=-\nabla q-T{\left(\frac{\partial p}{\partial T}\right)}_{\rho }\left(\nabla \cdot v\right)+\tau :\nabla v$$

where $\frac{D}{Dt}$ represents the material derivative, ρ is the fluid density, and ∇, v,g, *P*, $C_{v}$, and *T* denote the Hamilton operator, velocity vector, density, gravitational field, pressure, heat capacity ratio, and temperature, respectively. τ is the extra stress tension defined by a constitutive model. In this work, the rheological behavior of the PP melt is described in terms of the Giesekus model, as it yields a realistic behavior for melt flows, and the model description requires few parameters [51,52,53]. The constitutive equation is written as

(4) $$\tau =\tau_{P}+\tau_{S}$$

(5) $$\tau_{P}+\lambda \frac{\alpha }{\eta_{P}}\left(\tau_{P}\cdot \tau_{P}\right)+\lambda {\overset{\nabla }{\tau }}_{P}=2\eta_{P}D$$

(6) $$\tau_{S}=2\eta_{S}D$$

(7) $$D=\frac{1}{2}\left(\left(\nabla v\right)+{\left(\nabla v\right)}^{T}\right)$$

where $\eta_{S}\;\mathrm{and}\;\eta_{P}$ represent the Newtonian contribution viscosity and viscoelastic polymeric contribution, respectively, D is the deformation rate tensor, and ${\overset{\nabla }{\tau }}_{P}$ is the upper-convected time derivative of $\tau_{P}$.

For the gas, the constitutive equation is

(8) $$\tau =2\eta D_{ΙΙ}\;\mathrm{with}\;D_{ΙΙ}=\frac{1}{2}\left(\left(\nabla v\right)+{\left(\nabla v\right)}^{T}\right)-\frac{1}{3}\nabla v\delta_{ΙΙ}$$

where $\delta_{ΙΙ}$ is a second-order unit tensor.

#### 2.2.4. Boundary Conditions and Simulation Parameters

With the calculation domain shown in Figure 5, the boundary conditions are set as follows:

AB: Inlet of outer gas, a flow rate inlet; $Q_{oi}=7.5\times 10^{-5}m^{3}/s$, T = 473 K.

BC: Inlet of PP melt, a flow rate inlet; $Q_{melt-in}=1\times 10^{-7}m^{3}/s$, T = 473 K.

CD: Inlet of inner gas, a flow rate inlet, with flow rate (referred to as “gas flow rate in inner gas-assisted layer”, or “inner gas flow rate” for short hereinafter) values set corresponding to those in the extrusion experiments, i.e., inner gas flow rate $Q_{ii}=5\times 10^{-5}m^{3}/s$, T = 473 K.

GH: Outlet of outer gas, a pressure outlet; p=0 (compared with atmospheric pressure).

MN: Section of PP melt selected for calculation; p=0.

NO: Section of inner gas selected for calculation, set as a pressure outlet. The value was equal to the drop in pressure of gas from this section to the end face of the PP micro-tube (natural flow outlet of inner gas) and can be written in the form $p=\frac{32}{Re}\frac{l}{d}\rho {\bar{w}}^{2}$ with Re=ρw¯dμ [54], where $d,\rho ,\bar{w,}$ and μ are the inner diameter of the micro-tube, density, mean velocity, and kinetic viscosity, respectively, and l is the distance from the section selected for calculating the end face of the PP micro-tube. In this study, its value was taken as half the distance between the die exit and the traction device.

EF: Forced exhaust outlet of inner gas, set as a flow rate outlet with four different values. The values respectively corresponded to those in the four extrusion experiments, i.e., forced exhaust flow rates of $Q_{eio}=0m^{3}/s$,$\;2\times 10^{-5}m^{3}/s$,$\;4\times 10^{-5}m^{3}/s$, and $5\times 10^{-5}m^{3}/s$, and they were named case 1, case 2, case 3, and case 4, respectively.

AG, DJKE: Zero wall, T=473K.

BH, CN: Interfaces between the gas and melt.

HM: Free surface.

FO: Axis of symmetry.

Simulation parameters: The constitutive model’s parameters [55] used in the numerical simulation are shown in Table 4.

#### 2.2.5. Grid Resolution Effects

**Figure 6:**
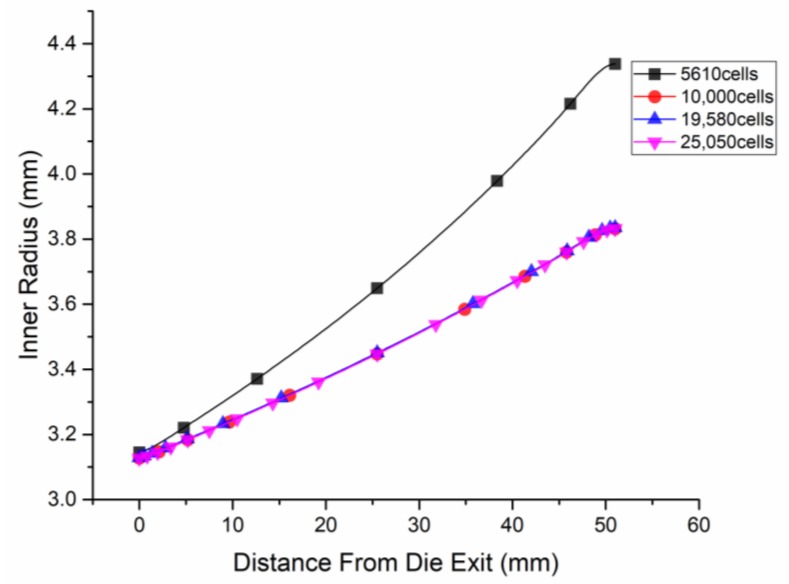
Different grid resolutions on analysis results of the inside radius.

To test the influences of different grid resolutions on the analysis results, we ran three additional cases on grids that had varying levels of refinement, and these additional grids had 5610, 19,580, and 25,050 cells, respectively. The examples depicted here had an inlet flow rate of $Q_{ii}=5\times 10^{-5}m^{3}/s$ at boundary CD, and the forced exhaust flow rate at boundary EF was $Q_{eio}=3\times 10^{-5}m^{3}/s$. Then, the inside radius of the micro-tube for these meshes was compared, as presented in Figure 6. The case with 10,000 cells had nearly the same result as the other two cases with more cells and was clearly different from the case with 5610 cells. As a result, the 10,000-cell grid was deemed significant enough and was used in subsequent studies.

## 3. Results and Discussion

This study focuses on the relationship between the change in boundary conditions at the exit of inner-assisted gas and the change in the radial size of the PP micro-tube. The discussion of the experimental and calculated results focuses on the flow of melt and inner-assisted gas in the area outside the die, downstream of the die exit.

### 3.1. Radial Dimension Distribution

**Figure 7:**
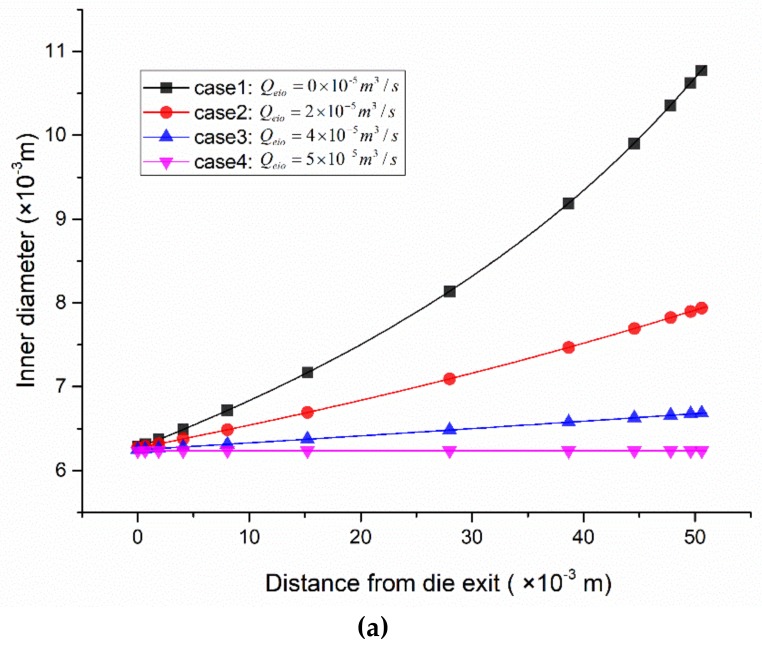

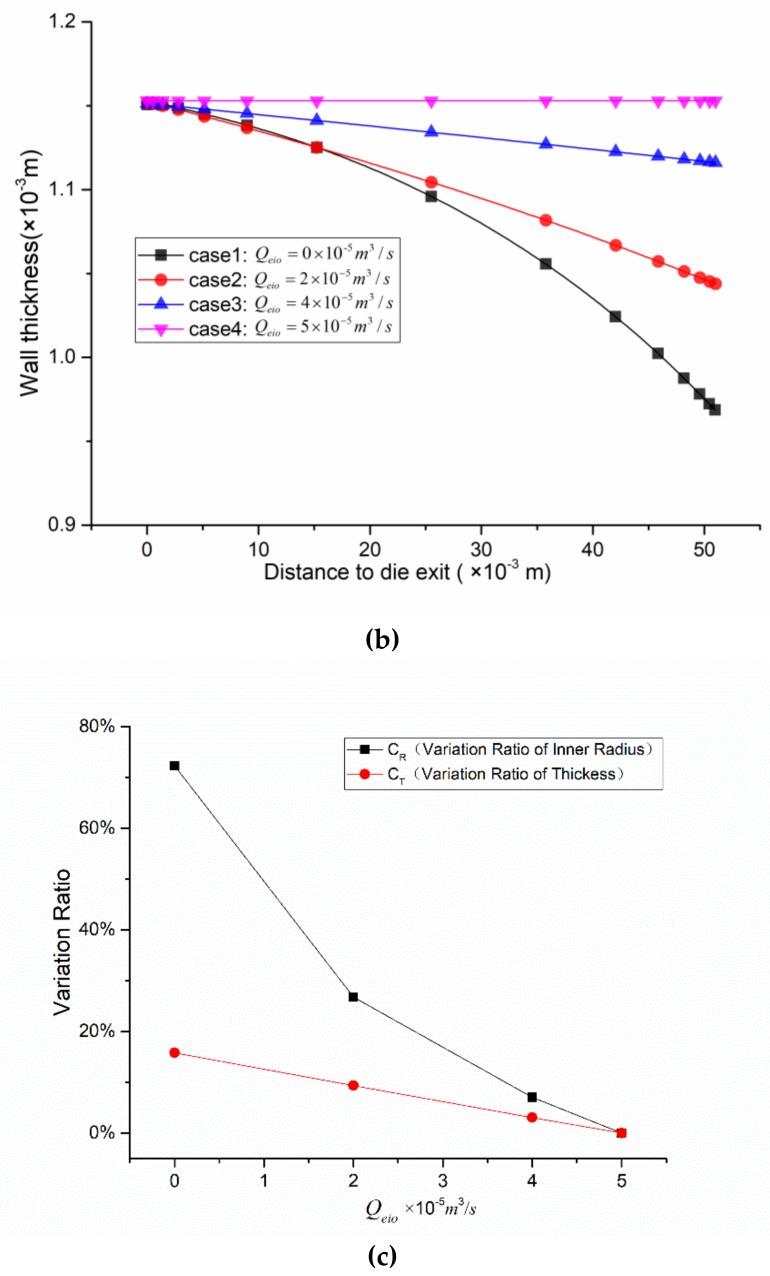
Micro-tube radial dimension distribution. (**a**) Inner diameter along the axial direction; (**b**) the thickness along the axial direction; (**c**) variation ratio of the inner diameter and thickness versus the forced exhaust flow rate.

**Figure 8:**
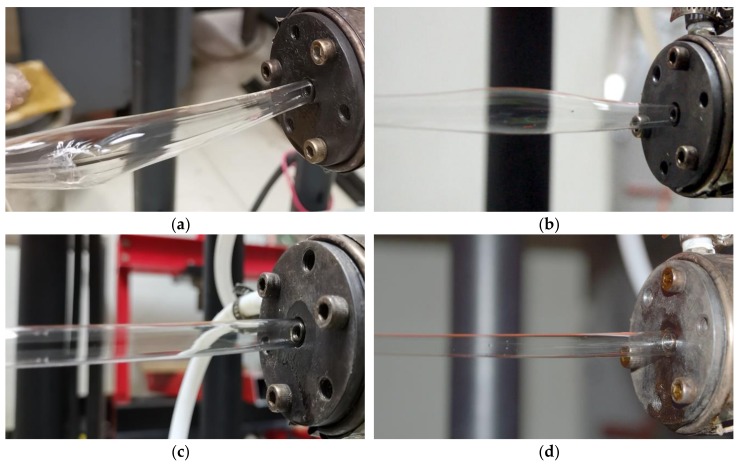
Results of extrusion formation for PP micro-tubes under different forced exhaust flow rates. (**a**) Forced exhaust flow rate $Q_{eio}=0m^{3}/s$; (**b**) forced exhaust flow rate $Q_{eio}=2\times 10^{-5}m^{3}/s$; (**c**) forced exhaust flow rate $Q_{eio}=4\times 10^{-5}m^{3}/s$; (**d**) forced exhaust flow rate $Q_{eio}=5\times 10^{-5}m^{3}/s$.

In this study, the morphology of PP micro-tubes was evaluated by the inner diameter and wall thickness. The PP micro-tube inner diameter variation ratio was $C_{R}=\frac{D_{N}-D_{I}}{D_{I}}\times 100\%$ and the wall thickness variation ratio was $C_{T}=\frac{T_{N}-T_{I}}{T_{I}}\times 100\%$, where $D_{N}$ and $T_{N}$ are the inner diameter and wall thickness at the calculated section (at boundary MN) and $D_{I}\;\mathrm{and}\;T_{I}$ are the inner diameter and wall thickness at the die exit section (at boundary HI), respectively. The calculation results are shown in Figure 7 and Figure 8. Figure 7a,b respectively depict the inner diameter variation and wall thickness variation of the PP micro-tube along the axial direction when the forced exhaust flow rate $Q_{eio}$ takes different values. Figure 7c describes the trend of the inner diameter variation ratio and wall thickness variation ratio with the change in the forced exhaust flow rate. Figure 8 illustrates the extrusion results of micro-tubes at four different forced exhaust flow rates.

It can be seen from Figure 7a,c that when the forced exhaust flow rate is smaller than the inner gas flow rate, the inner diameter of the micro-tube increases as the distance from the die exit increases, the inner diameter variation ratio decreases with the increase in the forced exhaust flow rate, and the rate of decrease shows a nonlinear trend from fast to slow. When the forced exhaust flow rate is increased to be equal to the inner gas flow rate, the expansion of the micro-tube almost disappears, and the inner diameter variation ratio obtained by numerical calculations is reduced to 0.016%, which is approximately equal to zero.

From Figure 7b,c, it can be seen that when the forced exhaust flow rate is lower than the inner gas flow rate, the wall thickness of the micro-tube decreases as the distance from the die exit increases; the thickness variation ratio decreases as the forced exhaust flow rate increases, and the decrease rate varies linearly. When the forced exhaust flow rate is increased to be equal to the inner gas flow rate, the wall thickness variation ratio of the numerical calculation results is reduced to 0.005%. At this time, the wall thickness of the micro-tube outside the die remains almost unchanged.

Figure 8 corresponds to the extrusion experiments at four different forced exhaust flow rates.

In Figure 8a, the forced exhaust flow rate is 0 $m^{3}/s$ after the gas from the inner gas-assisted layer exits the die, and it flows into the atmosphere through the lumen along the extrusion direction. Due to the small size of the lumen, the gas cannot flow away in a timely manner, causing the micro-tube to inflate and eventually rupture. Also, it was found that the swelling and rupture occurred intermittently and periodically, with an interval between adjacent rupture points of about 1.2 m.

In Figure 8b, the forced exhaust flow rate is $2\times 10^{-5}m^{3}/s$. That is, after the gas from the inner gas-assisted layer flows out of the die, a small part passes through the airway in the mandrel and is pumped into the atmosphere by the vacuum pump. Most of the rest still flows into the atmosphere naturally through the lumen along the extrusion direction. During this process, the micro-tubes also swell periodically, but no rupture occurs. The distance between adjacent swelling points is about 1.9 m.

In Figure 8c, the forced exhaust flow rate is $4\times 10^{-5}m^{3}/s$. That is, most of the inner assisted gas is exhausted from the airway in the mandrel, and the rest naturally flows into the atmosphere through the lumen along the extrusion direction. During this extrusion process, it can be observed that the micro-tube is blown uniformly (similar to that in blown film processing, the micro-tube blank is blown into a uniform film bubble), and there is no apparent periodic expansion node and no fracture.

In Figure 8d, the forced exhaust flow rate is $5\times 10^{-5}m^{3}/s$. All of the inner assisted gas is exhausted from the airway in the mandrel. Under these processing conditions, throughout the entire process―from being squeezed out of the die to entering the cooling device—the micro-tube does not swell; its diameter remains uniform. This is consistent with the results of numerical calculations. 

Combined with the four cases described in Figure 8, it can be found that the stable inner and outer gas-assisted layers form inside the die, and no die swell is observed at the die exit. Meanwhile, after being squeezed out of the die, as the forced exhaust flow rate varies, the micro-tube morphology becomes significantly different. Compared with Figure 7, the trend of this change is consistent with that of the numerical simulation results. It also can be inferred that the forced exhaust flow rate variation only affects the morphology of the PP micro-tube after it is squeezed out of the die, and it does not affect the flow state of the gas and PP melt inside the die.

In order to further evaluate the dimensional stability of the manufactured products, we cut out a 2-meter-long section from the tube obtained with forced exhaust flow (the forced exhaust flow rate was $5\times 10^{-5}m^{3}/s$), took a measurement point every 0.5 m from the beginning to the end, and measured the tube diameter and wall thickness at these five points. The measured data are listed in Table 5. The outer diameter of the tube was in the range of 7.91–8.01, and the wall thickness was in the range of 0.94–1.04, showing a small change in size. 

### 3.2. Melt Velocity Distributions

**Figure 9:**
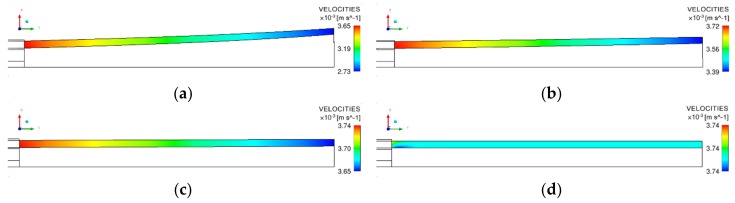
Velocity nephogram of melt downstream of the die exit. (**a**) Forced exhaust flow rate $Q_{eio}=0m^{3}/s$; (**b**) forced exhaust flow rate $Q_{eio}=2\times 10^{-5}m^{3}/s$; (**c**) forced exhaust flow rate $Q_{eio}=4\times 10^{-5}m^{3}/s$; (**d**) forced exhaust flow rate $Q_{eio}=5\times 10^{-5}m^{3}/s$.

**Figure 10:**
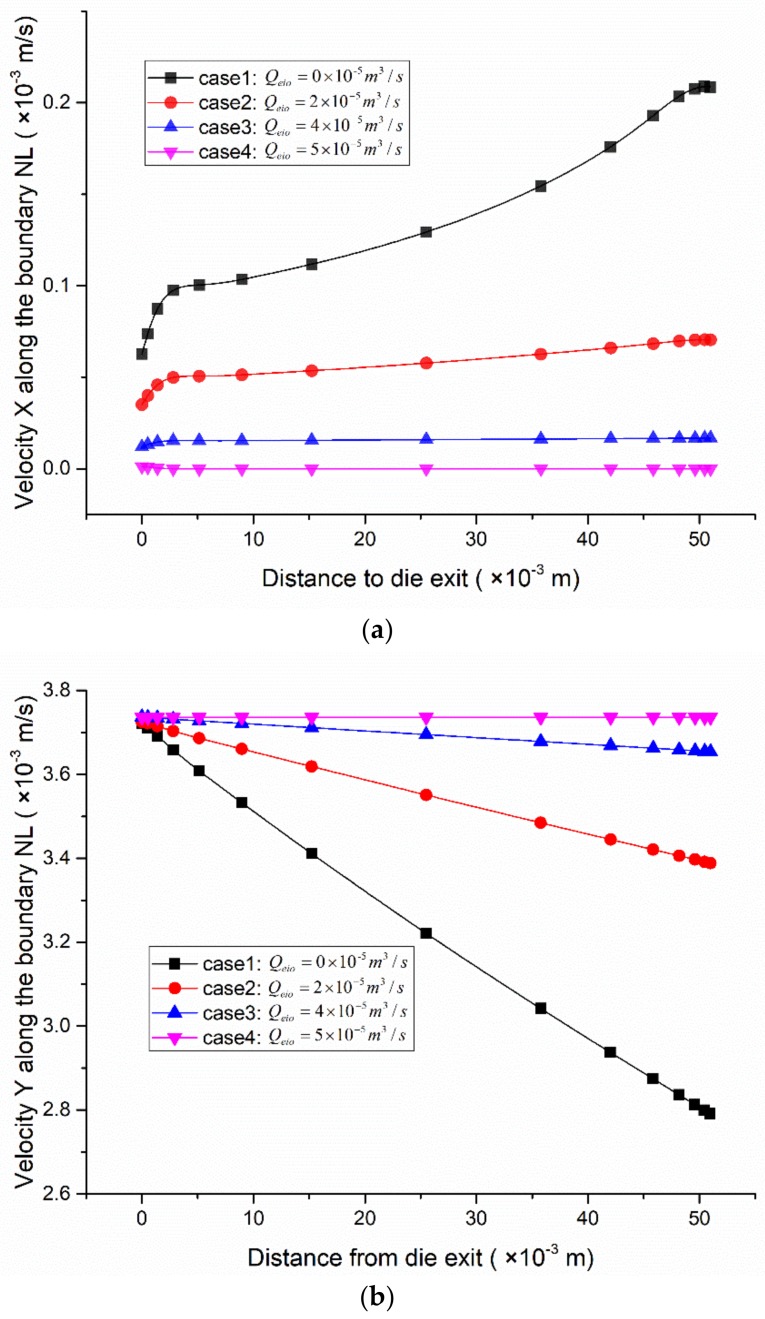
Velocity distributions along the interface between the inner gas and the melt. (**a**) Distributions of velocity X; (**b**) distributions of velocity Y.

Figure 9 is the velocity nephogram of the axial section of the micro-tube, and Figure 10 shows the velocity distribution in the X-direction and Y-direction on the micro-tube’s inner surface. It can be seen from Figure 9 that under a specific forced exhaust flow rate, after being squeezed out of the die, with an increase in the distance from the die exit, the micro-tube’s flow velocity decreases gradually, and the rate of decrease slows down with an increase in the forced exhaust flow rate. 

Combined with Figure 10, further analysis of the velocity distribution on the micro-tube inner surface (on boundary NL in Figure 5) showed two pieces of information. First, when the forced exhaust flow rate changes, at the same distance from the die exit, velocity X decreases with an increase in the forced exhaust flow rate. This shows that the inner diameter variation ratio decreases with an increase in the forced exhaust flow rate (Figure 7, Figure 8). Second, under a certain pumping rate, with an increase in the distance from the die exit, the velocity of the X component gradually increases, while that of the Y component decreases. The increase in velocity X causes the micro-tubes to expand continuously (Figure 7) in the radial direction. The expansion of the micro-tube diameter and the incompressibility of the melt explain the change in velocity Y.

### 3.3. Gas Pressure distributions

**Figure 11:**
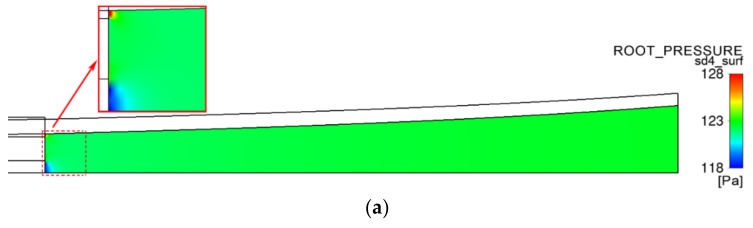

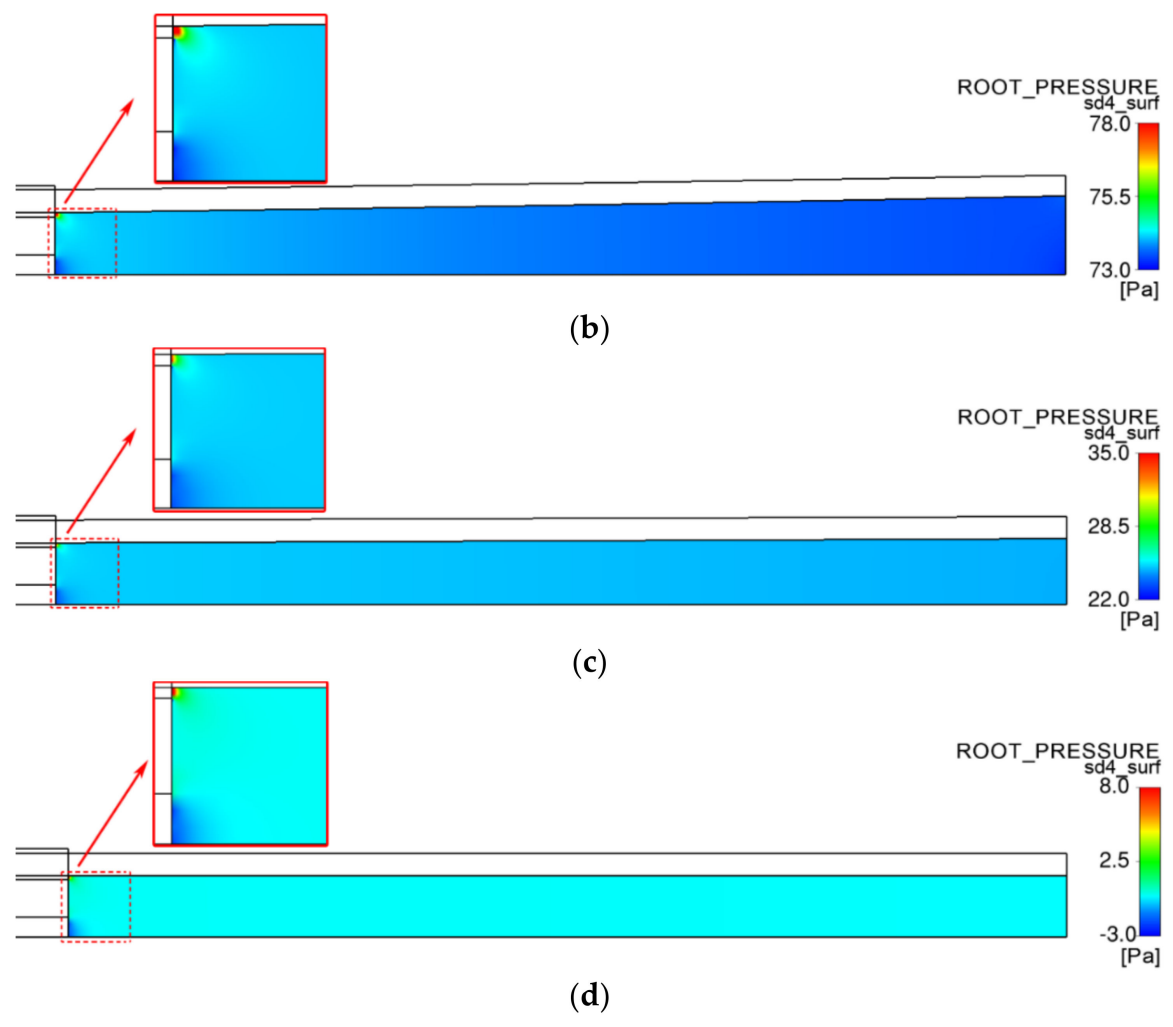
Pressure distributions in the PP micro-tube lumen. (**a**) Forced exhaust flow rate $Q_{eio}=0m^{3}/s$; (**b**) forced exhaust flow rate $Q_{eio}=2\times 10^{-5}m^{3}/s$; (**c**) forced exhaust flow rate $Q_{eio}=4\times 10^{-5}m^{3}/s$; (**d**) forced exhaust flow rate $Q_{eio}=5\times 10^{-5}m^{3}/s$.

Figure 11 shows the pressure nephogram in the lumen of the micro-tubes, from which it can be seen that the gas pressure distribution in the micro-tube has a level of regularity.

The maximum value of pressure in the lumen is located at the inner assisted gas outlet on the end face of the die. This is because the inner diameter of the micro-tube is about 100 times the thickness of the inner gas-assisted layer. After exiting the die, the gas rapidly expands in the lumen, and the pressure decreases accordingly. The minimum pressure level occurs at the outlet at the end face center of the mandrel, and this is due to the gas being forcibly exhausted from the lumen through this outlet, where the gas flow velocity is maximal, so the corresponding pressure is minimal.

When the forced exhaust flow rate is smaller than the inner gas flow rate, the air pressure in the micro-tube lumen is greater than zero; that is, the pressure difference between the inside and outside of the micro-tube is positive. Due to the effect of this pressure difference, the micro-tube wall has an acceleration pointing out of the micro-tube, so the wall velocity changes in the radial direction (Figure 10a), which also explains the root cause of the radial expansion of the micro-tube.

As the forced exhaust flow rate increases, the pressure in the lumen gradually decreases, and the expansion acceleration of the micro-tubes caused by the pressure difference decreases accordingly, which explains why velocity X of the micro-tube wall in Figure 10a increases as the forced exhaust flow rate decreases.

While the forced exhaust flow rate is equal to the inner gas flow rate, the air pressure in the micro-tube lumen is zero, except for in two particular areas—the micro area near the inner assisted gas outlet at the end face of the die and the micro area near the air forced exhaust port on the end face center of the mandrel. In this case, there is no longer a pressure difference between the inside and outside of the micro-tube, and there is no radial acceleration on the micro-tube wall. The content described in Figure 7 and Figure 8 is explained as follows: when the forced exhaust flow rate is equal to the inner gas flow rate, after the micro-tubes are squeezed out of the die, no radial expansion occurs.

## 4. Conclusions

Under the influence of flow resistance in the micro-tube lumen, after the inner assisted gas flows into the micro-tube lumen from the die, its pressure cannot be quickly reduced to atmospheric pressure, which causes a pressure difference between the inside and outside of the micro-tube wall. The existence of this pressure difference causes the micro-tube to exhibit radial expansion acceleration, which causes the diameter of the micro-tube that has not been completely solidified to become larger and the wall thickness to be thinner. Studies have shown that there is a positive correlation between micro-tube deformation and the gas flow rate in the micro-tube. To solve the problem of expansion and rupture of the micro-tube, it is necessary to start by regulating the gas flow rate in the micro-tube lumen.

Under the premise of not affecting the stability of the inner and outer assisted gas cushion, it is feasible and practical to regulate the gas flow in the micro-tube with this method. An independent airflow channel is manufactured in the mandrel. In the lumen from the inner assisted gas layer, the gas cannot flow into the atmosphere in time through the micro-tube lumen, so it is forcibly evacuated to the atmosphere through this dedicated channel. The lumen is thus no longer the main channel for the inner assisted gas to flow into the atmosphere. At the same time, the results also show that in order to eliminate the expansion and fracture of micro-tubes, it is necessary to set the forced exhaust flow rate equal to the inner gas flow rate. Under this condition, the double-layer gas-assisted extrusion can be stable and continuous, and micro-tubes with a constant radial dimension and sufficient length can be obtained.

## 5. Patents

Chinese patent: A gas-assisted extrusion die for microtubes (NO. 201920759010.1).

## Figures and Tables

**Table 1:** Property values for polypropylene.

Grade	$\mathrm{Density}\;\;kg/m^{3}$	Isotactic Index	Melt Flow Index (ISO1133) g/10 min	Activation Energy of Viscous Flow kJ/mol	Melting Temperature °C	Degradation Temperature °C
PPH-T03	900	≥96%	3	16.628	164–170	> 250

**Table 2:** Details of experimental apparatuses.

Apparatus	Specifications and Technical Parameters	Manufacturer
Extruder	GRQ-25PVC, screw diameter: 25 mm, L/D ratio: 25, type of feeding zone: gravimetric	HUAXI Plastic Machinery Co., Ltd., Dongguan, China
Cooling unit	GRQ-25PVC	
Haul-off device	GRQ-25PVC	
Air compressor	LD-800×3-80L, maximum gas exhaust volume: 405 L/min, max output pressure: 0.7 MPa	Juba Co., Taizhou, China
High-pressure gas storage tank	0.4 m^3^, Pressure limit: ≤1 Mpa	Dingxin Pressure Vessel Co., Ltd., Shenzhen, China
Dry filter	JB-01	Juba Co., Taizhou, China
Gas heating device (including temperature controller)	0.6 m^3^/h, 1000 W	Shanghai Laiheng Electric Appliance Co., Ltd., Shanghai, China
Pressure-adjustable valve	IR1000-01BG, Pressure regulation range: 0.05–0.2 Mpa	BLCH Pneumatic Science & Technology Co., Ltd, Yueqing, China
Throttle valve	ASC100-06,Allowable pressure range: 0.05–0.95 Mpa	Guangdong AirTAC Intelligent Equipment Co., Ltd., Shantou, China
Rotameters	LZB-40	Yinhuan Flowmeter Co., Yuyao, China
Pressure gauge	GS-40, Range 0–0.4 Mpa	Guangdong AirTAC Intelligent Equipment Co., Ltd., Shantou, China

**Table 3:** Processing parameters of the GAE forming experiments.

	Case 1	Case 2	Case 3	Case 4
The forced exhaust flow rate ($m^{3}/$s)	0	$2\times 10^{-5}$	$4\times 10^{-5}$	$5\times 10^{-5}$
The flow rate in the inner gas-assisted layer ($m^{3}/s$)	$5\times 10^{-5}$	$5\times 10^{-5}$	$5\times 10^{-5}$	$5\times 10^{-5}$
The flow rate in the outer gas-assisted layer ($m^{3}/s$)	$7.5\times 10^{-5}$	$7.5\times 10^{-5}$	$7.5\times 10^{-5}$	$7.5\times 10^{-5}$
Haul-off speed (r/min)	2	2	2	2
Extruder speed (r/min)	4	4	4	4
Die temperature (°C)	205	205	205	205
Melt temperature (°C)	205	205	205	205
Gas temperature (°C)	210	210	210	210

**Table 4:** Parameters calculated in the constitutive models.

Parameters	Total Viscosity (Pa·s)	Relaxation Time (s)	α	β=ηsη0
Melt	6624	0.1	0.3125	0.05485
Gas	2.6 × 10^−5^	-	-	-

**Table 5:** Dimensional data of the tube obtained with forced exhaust flow.

Measuring Point Number	1	2	3	4	5
Measuring point position	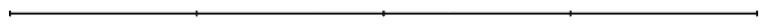				
Max/min outer diameter (mm)	7.97/7.93	7.99/7.94	7.97/7.93	8.01/7.91	7.99/7.92
Mean outer diameter (mm)	7.95	7.965	7.95	7.96	7.955
Max/min wall thickness (mm)	1.02/0.96	1.04/0.93	1.03/0.95	1.04/0.94	1.03/0.94
Mean wall thickness (mm)	0.99	0.985	0.99	0.99	0.985
